# Open-label placebos enhance test performance and reduce anxiety in learner drivers: a randomized controlled trial

**DOI:** 10.1038/s41598-024-56600-6

**Published:** 2024-03-20

**Authors:** Michael Schaefer, Sören Enge

**Affiliations:** Medical School Berlin, 12247 Berlin, Germany

**Keywords:** Open-label placebo, Placebo, Anxiety, Test anxiety, Distress, Psychology, Human behaviour

## Abstract

Passing the driving school test can be very challenging, especially in big cities, where up to 52% of all students fail this test. Consequently, many learner drivers experience stress and anxiety. For some learner drivers these feelings can be extreme and negatively affect the performance in the driving test. Different strategies to face anxiety and stress are known, including, for example, psychological or pharmacological approaches and even placebo pills. Recent intriguing findings have also demonstrated that placebos without deception, so-called open-label placebos, successfully reduce anxiety. Here we aimed to test effects of this novel treatment for learner drivers. We investigated whether open-label placebos affect test performance and feelings of anxiety in learner drivers. Sixty-eight healthy participants (mean age 21.94 years, 26 females) were randomized into two groups. The open-label placebo group received placebo pills two weeks before the driving test (two pills each day). The control group received no treatment. Results revealed that the open-label placebo group experienced significantly less anxiety than the control group before the test (measured with the State-Trait-Anxiety-Inventory, STAI-S, and the German Test Anxiety Inventory, PAF). Moreover, in the open-label placebo group less learner drivers failed the driving test (29.41% vs. 52.95%). The results suggest that open-label placebos may provide an ethical unproblematic way to experience less anxiety and might also enhance the probability to pass the driving test. We discuss possible mechanisms of open-label placebos and limitations of our findings.

## Introduction

In modern life, young people have to pass many written or oral exams at school or university. For students these tests are regularly accompanied by emotional distress, including anxiety, negative feelings or depressed mood, which in turn can negatively affect the performance of tests^1^. It has been reported that 13% of the first-year students suffer so much from test anxiety that they ask for help^2^. However, even beyond the academic environment there are challenging tests to pass, for example, when we apply for a new position and have a job interview or when trying to pass the driving school exam.

Test anxiety can be characterized by emotional distress and has affective, physiological, motivational, and cognitive facets^1,3,4^. Different strategies are known to face test anxiety, for example, psychological or pharmacological approaches^1^. Psychological approaches such as cognitive-behavioral skills training or mindfulness-based interventions try to use the cognitive facets to address anxiety^1^. Pharmacological strategies aim to reduce test anxiety by different drugs, for example benzodiazepines or other substances that enhance the activity of the neurotransmitter GABA (Gamma aminobutyric acid) and induce feelings of calm (but are also known to include the risk of addiction)^5^. Studies also revealed that placebos can be used to effectively reduce symptoms of anxiety and stress^6,7^. For example, Darragh et al. found that a take-home placebo treatment successfully reduced stress and anxiety in a non-patient population^8^. Balodis et al. examined stress-reducing placebo effects using the Trier Social Stress Test and found that placebos made participants to report less tension and anxiety, accompanied by a smaller increase in cortisol compared with a control group^9^.

Unfortunately, using placebos in a deceptive manner is associated with significant practical and ethical problems, since, for example, trust between patient and healthcare provider can be seriously damaged. Therefore, medical doctors seem to prescribe in particular impure (placebos with pharmacological active substances, but not on the specific disease or condition^10^) rather than pure placebos (no pharmacological effects at all)^11–13^. Physicians usually do not inform their patients that they receive placebos (or pharmaceuticals serving as placebos), because deception is believed to be crucial to see any placebo effects. However, recent studies in the last decade have reported that even placebos without deception, so-called open-label placebos (OLP), may affect, for example, pain or measures of psychological well-being, both in clinical samples and healthy subjects^14,15^. Furthermore, several studies demonstrated that OLPs successfully affect subjective well-being and test anxiety in healthy university students before taking an exam^16–18^.

It remains to be cleared why OLPs have effects. While in conventional placebos expectation and classical conditioning have been described as mechanisms for placebo responses^19–22^, the potential role of those processes in OLP studies is less clear. For example, it is discussed controversially whether expectation is crucial to see any OLP effects^23–25^. While a recent network meta-analysis emphasized the importance of positive treatment expectations^26^, other studies did not find that baseline expectations raised the OLP effect or were necessary to see any OLP effects (e.g.,^27^).

Furthermore, a major problem to assess the efficacy of OLP treatments is the risk of a potential bias in the results. It is obvious that OLP paradigms cannot be blinded, thus, participants know the hypothesis of OLP studies. Hence, considering that most OLP studies use self-report data, it is difficult to rule out response bias. Furthermore, it is unclear whether an OLP treatment affects predominantly subjective rather than objective measures. Only very few studies tried to measure OLP effects using physiological or objective behavioral outcomes, with mixed results (e.g.,^28–32^). For example, a recent review including 17 non-clinical samples showed OLP effects for self-report rather than objective outcomes^15^.

At first glance, the lack of effects in objective outcomes seems to be supported by recent OLP findings on test anxiety. While the above mentioned OLP studies consistently reported an effect on psychological measures, only one study found an effect (significant at borderline) for test performance^18^. However, examining OLP effects on test performance at the university may be difficult. Since sometimes the failure rate at university exams is very low, a statistical ceiling effect may occur. In addition, statistical problems due to the type of scales are known, especially when using the specific German grading system (e.g., when calculating means). Moreover, there may be more general problems, for example, when including university students in medicine and psychology as participants. It is reasonable that both groups are well aware of placebo effects (and possibly even of open-label placebo effects). This may be particularly a problem for OLP studies, since the participants are not blinded and know the hypothesis. Furthermore, these samples consist predominantly of females. At least in conventional placebo studies the role of sex in placebo effects is discussed controversially^33,34^.

Thus, it remains unclear whether an OLP treatment affects only subjective measures such as (self-reported) test anxiety or also objective measures such as test performance. The present study tries to tie in there. We aimed to address these points by examining anxiety and test performance in learner drivers. This sample can be characterized by a high failure rate. In Germany, in big cities up to 52% fail this test (in total, 43% of learner drivers in Germany failed to pass the driving test in 2022, according to the German Kraftfahrt Bundesamt^35^), making ceiling effects less likely. In addition, to obtain a driving license in Germany is comparatively expensive. Thus, due to the high failure rate and high costs one can assume that participants feel test anxiety. Moreover, this population is more representative, since it consists not only out of (predominantly female) psychology and medicine students, who may be especially open-minded to placebo (and even OLP) effects^36^.

In order to test the efficacy of OLP effects on anxiety and performance we conducted a randomized controlled trial on driver learners two weeks before passing the driving test. We hypothesized that for the OLP group both test anxiety and performance developed better (less increase in anxiety, better test results) compared with the control group. A second aim of this study was to test whether expectation is linked to the OLP related changes. Therefore, we examined different expectation measures to investigate the role of this mechanism in OLP studies.

## Materials and methods

### Participants

**Figure 1:**
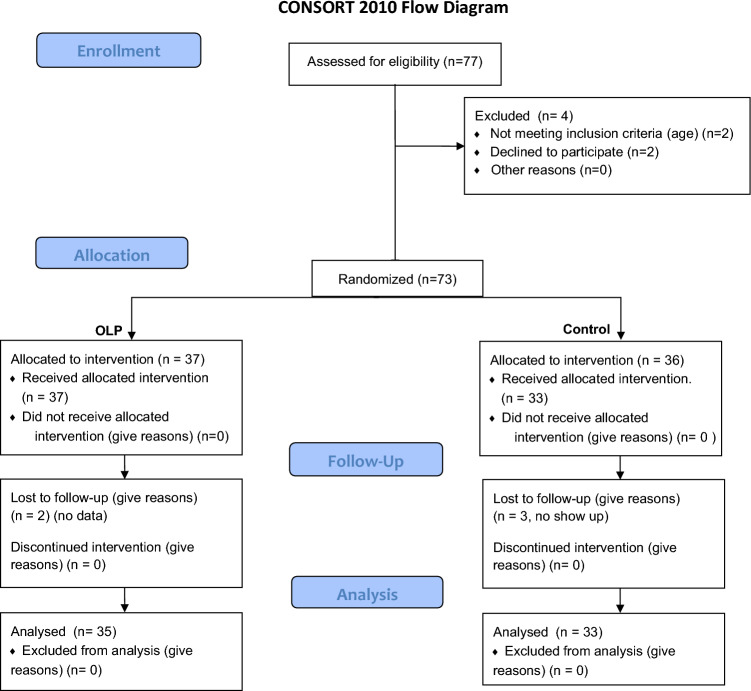
Flow diagram.

68 healthy learner drivers with no neurological or psychiatric history (self-reported) took part in the experiment (mean age 21.94, standard deviation ± 6.08 years, 26 females, see FlowChart, Fig. 1). The study adhered to the Declaration of Helsinki and was approved by the ethics committee of the Medical School Berlin. All participants provided written informed consent. The study was pregistered at the German Clinical Trail Registration (DRKS00031814, registered at 08/05/2023). Participants were recruited by contacting driving schools in Berlin, Germany. Inclusion criteria were being a student in a driving school and age between 18 and 60. Exclusion criteria were any known psychiatric or neurological history.

### Study design

Participants were allocated to a two-week randomized controlled trial comparing an OLP with a no-treatment control group before passing the practical part of the driving test. Driver learners were randomized into two groups. The OLP group received two placebos without deception each day for two weeks before passing the test. A control group received no pills. Test anxiety with respect to the driving test was examined at the beginning and on the day before the driving test.

Sample size was calculated based on previous OLP research^18^ using GPower^37^. Based on a desired power of 0.80, an effect size of 0.7, and an alpha error probability of 0.05 we computed a required minimum number of participants of 26 per cell. To account for possible dropouts, we aimed to enroll a total sample size of about 70 participants.

### Procedure

At the first visit participants completed baseline assessments including questionnaires with respect to anxiety feelings and to test anxiety. Moreover, we asked the participants to assess their test anxiety in general by using a visual analogue scale (VAS, a 10 cm long straight line with ends “not at all test anxiety” to “very strong test anxiety”). Then all participants were informed about the placebo effect as described previously^38^. We decided to inform all participants before assigning them to groups to keep the participant-healthcare provider relationship constant for all groups. This information stated that placebos are inactive substances and that they contain no medications, but placebo effects may still be powerful. Moreover, we explained that the body may automatically respond to taking placebo pills, that a positive attitude is helpful (but not necessary), and that placebo pills need to be taken faithfully. In addition, we added information that recent research showed that even placebos without deception have positive effects. Last, we showed a news video reporting OLP effects (analogue to^39^). Then we randomized the participants into two groups. Participants in the OLP group received 28 placebo pills with the instruction to take one pill in the morning and one for night (each day for two weeks). Placebo pills consisted out of cellulose microcrystalline in a vegetal capsule. They were packed in a white box labelled with the logo of the local university and marked with: “Placebo pills (28), take one in the morning and one in the evening, for 14 days”. Participants in the control group received no treatment but were explained about the importance of the control condition. All participants then received a short diary, in which they were asked to document each day the anxiety with respect to the practical driving exam (using VAS scales). After two weeks, on the day before the driving test, participants completed the second assessment, in which the participants again completed questionnaires about anxiety. Finally, participants informed us about the result of the driving test.

### Outcome measures

Primary outcome measure was the change in the German version of the State-Trait-Anxiety-Inventory (STAI-S, state version)^40,41^, which is an established questionnaire and widely used to exam anxiety feelings.

Secondary outcome measure addressed more specifically test anxiety, which was assessed with the German Test Anxiety Inventory (PAF^42^). This questionnaire has been used before to test anxiety in schools and college students and also in previous OLP studies (e.g.,^16,18^).

Furthermore, we examined explorative outcomes. Thus, we asked the participants after the driving test whether they succeeded or not. In addition, we examined the belief in placebos in different ways. To assess the general belief in placebos we used four items, which have been previously used^29^. To assess specifically the participant’s belief in open label placebos we used 5 questions embedded in more general questions (identical to^31^). Moreover, the participants were asked to rate their expectation about the success of the placebo treatment at baseline (VAS scale). Furthermore, at the end of the trial (on the day before the driving test) participants were also asked to assess their feeling whether they think the placebos had indeed reduced the felt anxiety (VAS scale, OLP group only). The control group was asked whether they were disappointed to have been randomized to the placebo group. Finally, all participants had to assess the perception of the experimenter (analogue to^31^).

### Randomization and blinding

Randomized group assignment was achieved by a computer-generated random number sequence. In contrast to the participants the experimenters were blinded to the group assignment (with the exception of the one assigning the group).

### Statistical analysis

To test our hypotheses, we computed ANCOVAs with change scores from pre to post (STAI and PAF) and baseline data (of the relevant variable) as a covariate^43^. In addition, we examined group differences at baseline (STAI, PAF, and other variables) to test whether there are any group differences before starting the trial. Diary data was analyzed by calculating a change score between the mean of the first and last 3 days (identical to^44^) and then computing an ANCOVA in an analogue way. Statistical analyses were performed with IBM SPSS Statistics (Version 27), using an alpha level of < 0.05.

## Results

Demographic details and baseline characteristics can be found in Table 1. Placebo and control groups did not differ with respect to baseline characteristics (e.g., anxiety, all *p* > 0.10). For example, at the beginning of the trial (baseline, two weeks ahead of the test) both groups stated to feel similarly anxious with respect to tests in general (OLP group: 5.86 ± 1.94, control group: 5.67 ± 2.22, on VAS 0–10 with 10 = very strong). Nine participants claimed to feel disappointed to be assigned to the control group.

**Table 1:** Demographics and baseline characteristics (mean ± standard deviations).

Characteristic	Open-label placebo	Control group	*p* value
N	35	33	
Age (in years)	22.40 ± 7.43	21.45 ± 4.41	
Females/males	14/21	12/21	
Failure in previous driving test	8	7	
PAF	48.65 ± 11.94	48.30 ± 10.23	*p* = 0.900
STAI	46.17 ± 10.24	46.52 ± 8.97	*p* = 0.884
General test anxiety (VAS, 0–10)	5.86 ± 1.94	5.67 ± 2.22	*p* = 0.707

*p* values refer to results of t-tests.

### Primary outcomes

**Figure 2:**
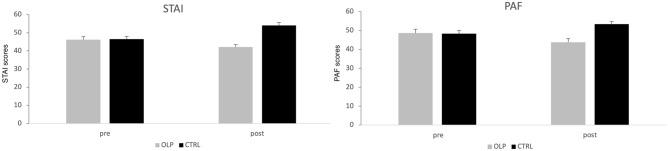
Outcome of anxiety questionnaires (STAI, PAF) for 2 weeks before driving test (means and standard errors). Results show higher anxiety after two weeks for the control group.

To test our hypotheses, we analyzed the change scores of the felt anxiety (STAI) from pre to post. Results revealed that anxiety in the control group increased over time, in contrast to the OLP group, which showed even less anxiety than during baseline (change scores OLP group: − 4.00 ± 7.33, control group: 7.55 ± 7.05). An ANCOVA with the baseline scores as covariate demonstrated a strong effect for the OLP treatment (F (1, 65) = 66.69, *p* < 0.001, partial eta^2^ = 0.51) (see Fig. 2). There were no correlations of this change with the belief in open placebos (r = 0.06), with the belief in placebos in general (r = − 0.08), or with the expectation at baseline that the treatment will work (r = 0.28) (Pearson, all *p* > 0.10).

### Secondary and explorative outcomes

**Figure 3:**
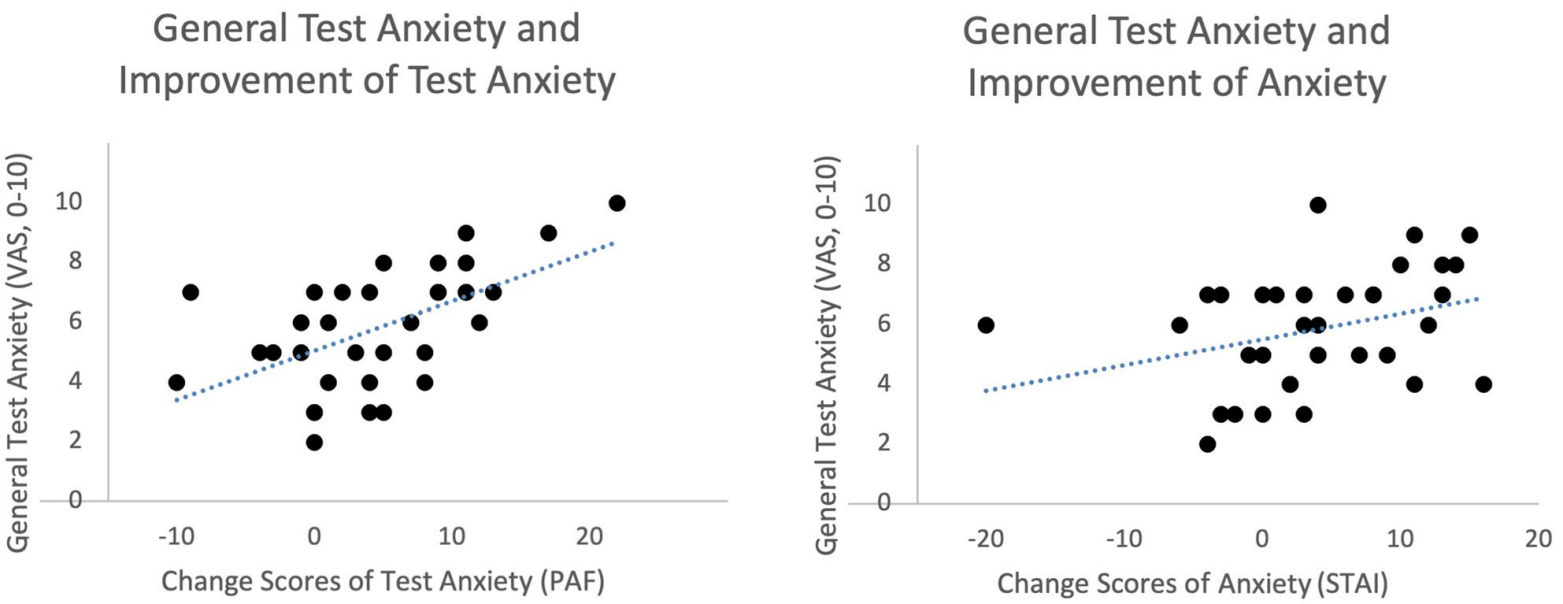
Scatterplot of general test anxiety (examined at baseline, VAS scores) and change scores of anxiety (STAI, on the right) and test anxiety (PAF, on the left) (OLP group only). Results demonstrated that participants with high test anxiety profited most from the OLP treatment. While the relationship between the general test anxiety (VAS) and the improvement of test anxiety (PAF) was significant (r = 0.57, *p* < 0.001), the correlation with the improvement in anxiety measured with STAI failed to reach the level of significance (r = 0.32, *p* = 0.057). However, when excluding the outlier on the left this correlation shows a significant relationship (r = 0.40, *p* = 0.018), too.

We further tested effects of the OLP treatment on test anxiety by comparing change scores of the PAF. Results showed a similar outcome. Test anxiety measured with the PAF increased for the control group over time but decreased for the OLP group (change scores OLP group: − 3.85 ± 6.20, control group: 5.06 ± 5.42). An ANCOVA revealed a strong effect for the OLP treatment (F (1, 64) = 49.87, p < 0.001, partial eta^2^ = 0.44). Again, we found no correlations of the amount of change with the belief in open placebos (r = − 0.03), with the belief in placebos in general (r = − 0.12), or with the expectation at baseline that the treatment will work (r = 0.21) (all *p* > 0.10). However, we found a correlation of the change scores with the baseline assessment of having high test anxiety in general (VAS score, r = 0.57, *p* < 0.001, see Fig. 3). There was also an analogue correlation of change scores in STAI with having high test anxiety in general, which was significant when removing an outlier (r = 0.40, *p* = 0.018).

We then examined whether the OLP treatment had affected the test exam. In the OLP group 29.41% of the learner drivers failed the driving exam, whereas in the control group 52.95% did not pass the test. However, this difference failed to reach the level of significance (Chi-square, *p* > 0.10). This might be explained by the limited number of participants for this test, since due to a technical error (data loss) only 34 participants went into this analysis (OLP group: 17, control group: 16). Nevertheless, when excluding 4 participants in the OLP group who claimed not to believe in OLPs (VAS score of less than 5 in OLP belief), results showed that in the OLP group significantly more learner drivers passed the driving test (Chi-square (Pearson): χ^2^(1) = 5.09, *p* = 0.024, Cramer’s V = 0.24).

**Figure 4:**
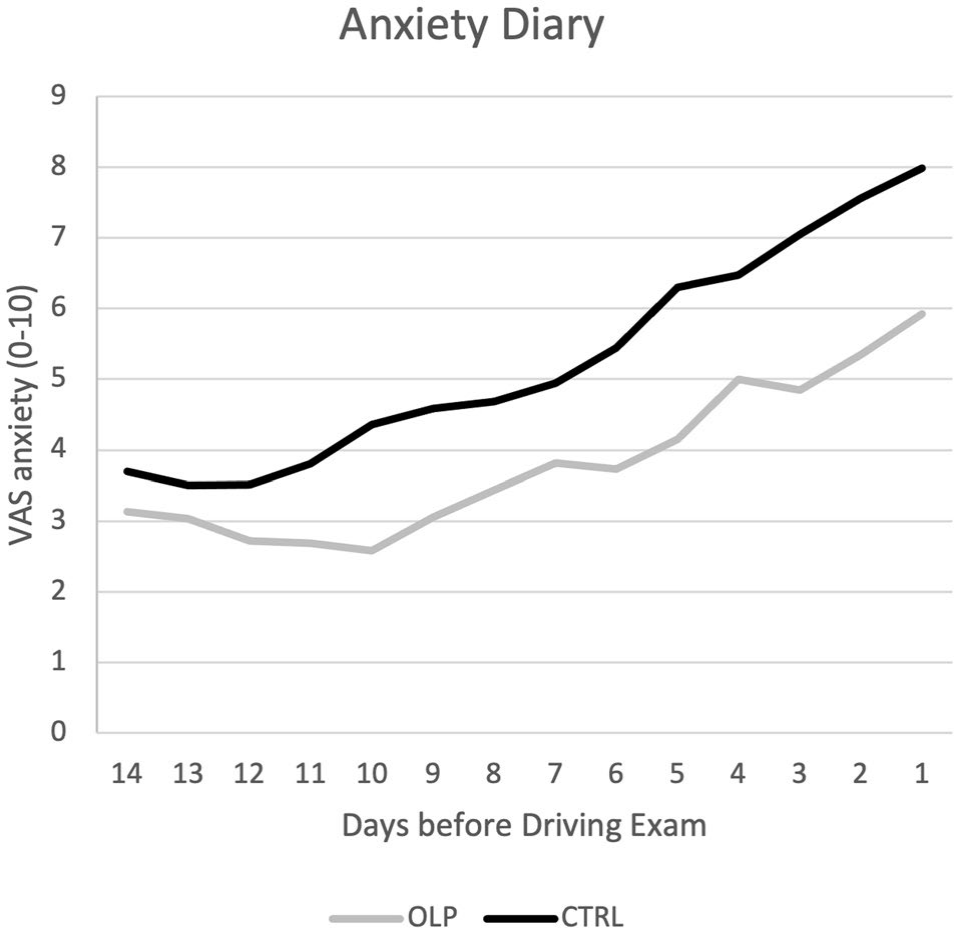
Outcome of diary data. Results show increase of anxiety scores (VAS) over time for both groups, but this increase was smaller for the OLP group.

Diary data on the felt anxiety supported the results of the STAI and PAF by showing that curves for OLP and control group developed differently, demonstrating that the OLP group felt less anxious than the control group after two weeks. This is also supported by a significant group effect when comparing the mean of the first three days with that of the last three days (F(1, 49) = 19.67, *p* < 0.001, partial eta^2^ = 0.29). However, in contrast to STAI and PAF the VAS scores of the diary data of the OLP group show that anxiety also increased for the OLP group, although this increase was significantly smaller than for the control group (change scores OLP: 2.65 ± 1.42; control group: 3.96 ± 1.70; see Fig. 4). The change in the diary data (increase of anxiety) was not predicted by the belief in OLPs (r = − 0.08) or placebos in general (r = − 0.17) or by the expectation that the treatment will work (r = 0.22) (all *p* > 0.10). Remarkably, expectation was linked to higher instead of lower anxiety outcomes.

**Figure 5:**
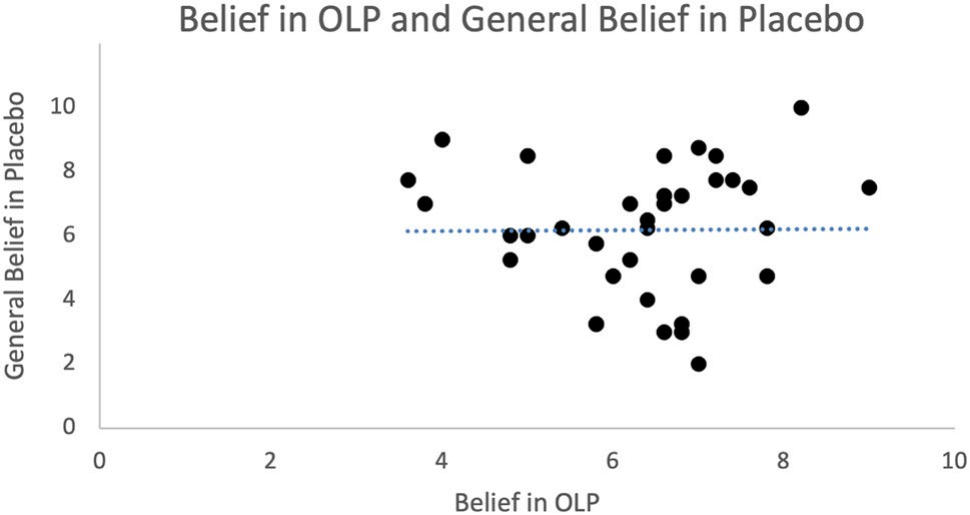
Scatterplot of belief in placebos in general and belief in OLPs. Correlation showed no significant relationship of these two measures.

When asking the participants of the OLP group whether they think that the placebo pills had reduced their anxiety (at the second measurement, on the day before the exam), participants claimed to confirm this statement (6.17 ± 1.22 on the VAS scale). However, this belief was not linked to the change scores in anxiety (PAF, STAI, diary data, all *p* > 0.10). Perception of the experimenter at the end of the trial was similar for both groups, no relationships of the change scores in anxiety with the perception of the experimenter were found. Similarly, the attitudes towards placebos in general were comparable for both groups. However, we found that participants of the OLP group believed stronger in OLPs than the control group (at the end of the trial, before the driving test, t (66) = 5.06, *p* < 0.01, see Table 2). Moreover, the more the participants felt that the nasal spray had worked, the more they were convinced by OLPs (r = 0.45, *p* = 0.007), although there was no relationship of OLP beliefs with improvements in anxiety scores measured with STAI and PAF, as outlined above. Finally, we tested whether the belief in OLP and the general belief in placebos were linked. Results showed no effects (*p* > 0.10, see Fig. 5).

**Table 2:** Results of assessment of placebo attitudes and perception of experimenter at the end of the trial (before driving test; mean ± standard deviations).

Characteristic	Open-label placebo	Control group	*p* value
Perception of experimenter	6.23 ± 0.43	6.41 ± 0.66	*p* = 0.398
Belief in OLP	6.33 ± 1.22	4.68 ± 1.44	*p* < 0.001
Belief in placebos in general	6.10 ± 2.01	6.02 ± 1.65	*p* = 0.717

*p* values refer to results of t-tests.

## Discussion

The current study aimed to test whether an OLP treatment would be successful to reduce anxiety and enhance the performance in the practical driving exam. Results showed that the OLP group reported less anxiety before the test compared with participants of the control group. Moreover, more learner drivers in the OLP group passed the driving test.

Our results are in line with recent studies on OLP effects in anxiety. Several studies reported that placebos without deception reduce anxiety and enhance psychological measures of well-being^16–18,32^. Previous studies found middle or high effect sizes for OLP effects on test anxiety. For example, a recent study on test anxiety in university students found effect sizes of d = 0.71^18^. The current study revealed even higher effect sizes of partial eta^2^ = 0.51, which is similar to effect sizes of OLP studies with clinical samples rather than healthy subjects^14,15^. Furthermore, the participants seemed to be convinced by the OLP treatment. Thus, using a more representative sample (with respect to gender and professions) and possible less bias (with respect to potential ceiling effects and knowledge of the participants about placebos) the results support previous results and demonstrate the efficacy of OLPs.

It is noteworthy that we found different results for diary data and results of the STAI and PAF questionnaires. Although we found effects for all three measures, diary data showed that participants of both groups felt more anxious shortly before taking the driving test compared with the beginning of the study, while STAI and PAF scores reported that OLPs made participants feel even less anxious before the test. What is the reason for this different picture? Differences with respect to the nature of these measures may account for the results. The diary data consisted out of VAS that had to be filled out for each single day. In contrast, STAI and PAF anxiety measures were completed at baseline and before the day of the exam. The diary consisted out of one single item asking for present anxiety with respect to the driving exam in a direct way, whereas questionnaires such as the STAI or PAF include many questions that tried to assess anxiety indirectly. However, the different results might also point to the problem of bias in OLP studies. Given that OLP studies cannot be blinded, one should be sensitive to this problem in this kind of studies. For example, since participants know the hypothesis of the trial, they might tend to (self) report their feelings in a way that they are consistent to this hypothesis. This might be especially a problem when completing the questionnaires at the end of the trial, because this may be a situation where the rationale of the study will be present to the participants. In contrast, the diary data is completed each day in a private situation, possibly making this kind of bias less likely. However, the lack of any correlations between the belief in OLPs (or other expectation measures) and anxiety changes in all three measures does not speak in favor to this explanation.

Nevertheless, it has to be stressed that in all three anxiety measures the OLP treatment significantly reduced anxiety feelings compared with the control group. The anxiety scores we report were based on self-report data. Previous work suggested that OLPs may work predominantly for subjective measures, whereas the impact of OLPs on objective measures is less clear (e.g.,^15^). Here we aimed to test this hypothesis by examining the test performance of the learner drivers. The practical driving test seems to be suitable because the failure rate is up to 52% (German Kraftfahrt Bundesamt^35^), which excludes ceiling effects and provides relatively strong anxiety feelings for the learner drivers. Indeed, we found that (at baseline) the learner drivers expressed clear anxiety feelings with respect to the driving test. Our results demonstrated that OLPs made it more likely to pass the driving test, thus, in our study OLPs also affected objective measures (although based on a limited sample size).

What are the mechanisms of OLPs? In conventional placebo studies expectation is thought to play a major role for placebo responses^19,20^, while the role for this mechanism in OLP studies is discussed controversially^20^. For example, it has been shown that baseline expectations predicted conventional placebo responses but not OLP effects in pain relief^27^. Therefore, it has been suggested that only double blind placebo effects may be based on prefrontal brain areas (which is thought to reflect expectation^20^), whereas OLP effects may be based on lower control mechanisms^45^. Several OLP studies support this view by demonstrating that expectation did not predict placebo responses (^23–25^ but see, e.g.,^26,46^). Hence, conventional double-blind placebos and OLPs may be based on different mechanisms^47^. This is also supported by a recent study showing different effects for the two placebo types when compared directly^44^. Our results are in line with this view by showing a lack of significant correlations of expectation measures with anxiety outcome changes (and even a negative relationship with diary data, see also^27^), suggesting that expectation is not the crucial mechanism for placebos without deception (see also^48^ for a general discussion of the role of expectation in placebos). None of our expectation measures (belief in OLPs, general belief in placebos, expectation at baseline, belief that treatment had worked) showed a significant correlation with the OLP related changes in anxiety. Remarkably, OLP belief and general belief in placebos were not linked. Thus, individuals who are convinced that placebo effects in general do occur, do not necessarily believe that placebos without deception might work as well.

If expectation is not the crucial mechanism for OLP effects here, how do placebos without deception work? Beyond classical conditioning or expectation some further theoretical assumptions have been discussed. Based on a Bayesian approach, the “Prediction and Error Processing” model states that we interpret new information with the background of recent experiences and thereby trying to calculate statistical predictions about the likelihood of the outcome of a (new) treatment such as OLPs^13,48^. Another approach refers to the term embodiment, which claims that mind and world interact via the body and thereby affect our cognitions. In contrast to classical conditioning, this theory does not require conditioning procedures^49–51^. Further studies are needed to shed more light on possible mechanisms of OLPs.

The present study shows that an OLP treatment successfully reduces anxiety before a test situation, along with better test results. We also found that the more the learner drivers suffered from test anxiety in general, the more they profited from the OLP treatment. This is in line with recent studies^46^, suggesting that OLPs with respect to anxiety are most promising in individuals with high anxiety feelings, and also with the general finding that OLP treatments seem to be stronger in patients than in non-clinical samples^14,15^.

Given that ethical arguments are less problematic in OLPs and effect sizes are remarkable, results on test anxiety might suggest OLPs for practical use. However, since we still do not know much about possible mechanisms, results should be treated carefully. In addition, several limitations apply to our trial. For example, the sample size is still limited (especially for the test outcome). Although the sample may be more representative with respect to some aspects, it still includes predominantly young individuals. Moreover, the limited sample size of the test outcome could be susceptible to bias. However, as the groups were evenly distributed, this may be less likely. In addition, although we considered results of a driving test as an objective measure, most measures of this trial are self-report data. Future studies should consider physiological data, e.g., cortisol measurements or brain imaging data. Furthermore, a comparison with conventional placebos would be very interesting. Only few studies include a comparison between conventional placebos with deception and OLPs^44,47,52,53^. In addition, a potential bias might be that participants of the control group were disappointed about their assignment. However, we found that only few participants stated to feel dissatisfied with their group allocation. Furthermore, using the diary may have affected the anxiety feelings itself. However, since both groups received this diary, we believe that it is unlikely that these effects systematically affected our results. Last, there are also several papers that could not replicate previous OLP findings (e.g.,^54,55^). Thus, we should be cautious when drawing conclusions out of these results, which need to be replicated by future studies.

Although these points need to be taken into account, we believe that the outcome of this study shows encouraging results. OLPs are cost-effective and ethical less problematic. They may help individuals not only in situations such as passing the driving test but also when facing similar challenges in daily life and potentially also patients in clinical samples.

### Supplementary Information

Supplementary Information.

## Data Availability

All data needed to evaluate the conclusions in the paper are present in the paper and/or the Supplementary Materials.
