# Supplementary material for: Open-label placebos enhance test performance and reduce anxiety in learner drivers: a randomized controlled trial
Source: Sci Rep. 2024 Mar 20;14:6684. doi: 10.1038/s41598-024-56600-6 (PMC10954622; doi:10.1038/s41598-024-56600-6)
Supplement: Supplementary file 1 — Supplementary Information. [file 41598_2024_56600_MOESM1_ESM.docx]

# Supplementary Material

**Open-label placebos enhance test performance and reduce anxiety in learner drivers: A randomized controlled trial**

**Michael Schaefer*, Sören Enge**

Medical School Berlin, 12247 Berlin, Germany

**S1:** Questions to assess expectation about the success of the treatment

To test whether participants expected that the placebo treatment will be successful, we asked participants “How strong is your expectation that the placebo treatment will reduce your test anxiety?” Participants indicated their answer by using a VAS scale with the ends “very low” and “very high”.

**S2:** Questions to assess effectiveness of placebo

To test whether participants believed that the placebo was effective in reducing their anxiety, we asked participants in the OLP group “Do you think the placebo nasal spray reduced your negative emotional response to the pictures?” Participants indicated their answer by using a VAS scale with the ends “not at all reduced” and “very much reduced”.

**S3:** Questions to examine general belief in placebos

These four items have been taken from Leibowitz et al. (2019). Participants rated the questions on a 11-point scale from 0 (definitely not true) to 10 (definitely true):

“Placebo effects are a part of all active medications.”

“Placebo effects can occur in all illnesses and conditions.”

“Placebo effects happen because the mind has the power to heal.”

“Placebo effects work because placebos influence people's expectations about a particular treatment.”

**S4:** Questions to examine belief in OLP

Five questions aimed to examine the belief in OLPs and were embedded in other more general belief items on pain. All questions are taken from Guevarra et al. (2020). Participants were asked to rate the statements on a 11-point scale from 0 (definitely not true) to 10 (definitely true).

“A placebo can still work on me even though I know that I am taking a placebo.”

“In order for placebos to work, the person needs to be deceived into believing they are taking an actual medicine.”

“A placebo can reduce my negative emotions even though I know I am taking a placebo.”

“A placebo only works if the person is deceived into thinking they are taking an actual medicine.”

“A placebo can reduce my pain even though I know that I am taking a placebo.”

**S5:** Questions to assess perception of the experimenter

To evaluate the perception of the experimenter participants rated the experimenter on the extent they were competent, knew what the experimenter was doing, authority, easy to understand, confident, likeable, warm, and cold on a 7- point Likert scale from 1 (= not at all) to 7 (extremely). One item was reverse coded. The items were taken from Guevarra et al. (2020).
